# The Pathway to Low Outlier Status in Venous Thromboembolism Events: An Analysis of Pancreatic Surgery in the National Surgical Quality Improvement Program

**DOI:** 10.1089/pancan.2020.0002

**Published:** 2020-06-29

**Authors:** Samantha L. Savitch, Tyler M. Bauer, Nkosi H. Alvarez, Adam P. Johnson, Theresa P. Yeo, Harish Lavu, Charles J. Yeo, Jordan M. Winter, Geno J. Merli, Scott W. Cowan

**Affiliations:** ^1^ Department of Surgery, Thomas Jefferson University, Philadelphia, Pennsylvania, USA.; ^2^ Jefferson Pancreas, Biliary, and Related Cancer Center, Department of Surgery, Thomas Jefferson University, Philadelphia, Pennsylvania, USA.; ^3^ Division of Internal Medicine, Thomas Jefferson University Hospital, Philadelphia, Pennsylvania, USA.

**Keywords:** venous thromboembolism, NSQIP, surgery

## Abstract

**Purpose:** Our institution's hepatopancreaticobiliary surgery service (HPBS) has demonstrated low rates of venous thromboembolism (VTE). We sought to determine whether the HPBS's regimented multimodal VTE prophylaxis pathway, which includes the use of mechanical prophylaxis, pharmacological prophylaxis, and ambulation, plays a role in achieving low VTE rates.

**Methods:** We compared pancreatic surgeries in the American College of Surgeons National Surgical Quality Improvement Program (NSQIP) participant user file with our institution's data from 2011 to 2016 using univariate, multivariate, and matching statistics.

**Results:** Among 36,435 NSQIP operations, 850 (2.3%) underwent surgery by the HPBS. The HPBS achieved lower VTE rates than the national cohort (2.0% vs. 3.5%, *p* = 0.018). Upon multivariate analysis, having an operation performed by the HPBS independently conferred lower odds of VTE incidence in the matched cohort (odds ratio = 0.530, *p* = 0.041).

**Conclusions:** We identified an independent correlation between the HPBS and decreased VTE incidence, which we believe to be due to strict adherence to and team participation in a high risk VTE prophylaxis pathway, including inpatient pharmacological prophylaxis, thromboembolic deterrent stockings, sequential compression devices, and mandatory ambulation.

## Introduction

Venous thromboembolism (VTE) is a hospital-acquired condition that has a significant impact on morbidity, mortality, and hospital costs. Between 100,000 and 180,000 deaths per year in the United States may be directly or indirectly related to all-cause VTE, with the total incidence being between 350,000 and 600,000.^1^ Approximately half of these are hospital-acquired VTE.^2^ Despite advances in diagnosis and treatment, VTE persists as a common cause of death in the inpatient setting.^3^

Although VTE continues to be a significant source of morbidity and mortality, there have been many safe and cost-effective interventions aimed at reducing VTE occurrence.^4^ Inpatient prophylaxis is multimodal and falls into three major categories: pharmacological, mechanical, and ambulatory. The most well-studied pharmacoprophylaxis medications are heparin and enoxaparin^5,6^; new direct oral anticoagulants have been primarily studied in joint replacement surgery and are also effective in preventing symptomatic VTE.^7^ Mechanical therapies, including thromboembolic deterrent (TED) stockings, and intermittent pneumatic compression (IPC) devices, have also been shown to decrease VTE risk.^8,9^ Finally, multiple studies have indicated that postoperative ambulation, along with concomitant pharmacological and mechanical prophylaxis, reduces the incidence of VTE, improves postoperative outcomes, and decreases length of stay (LOS) in thoracic, cardiac, abdominal, and orthopedic surgical patients.^10–12^ Furthermore, institutions with formal ambulation programs have reported enhanced adherence to ambulation protocols by patients and hospital staff.^13–16^ Many guidelines regarding VTE prophylaxis include explicit recommendations stating that hospitals and surgical services should synthesize a formal VTE prophylaxis algorithm that includes a risk assessment tool and a specified regimen for VTE prevention.^17–20^

**FIG. 1.**
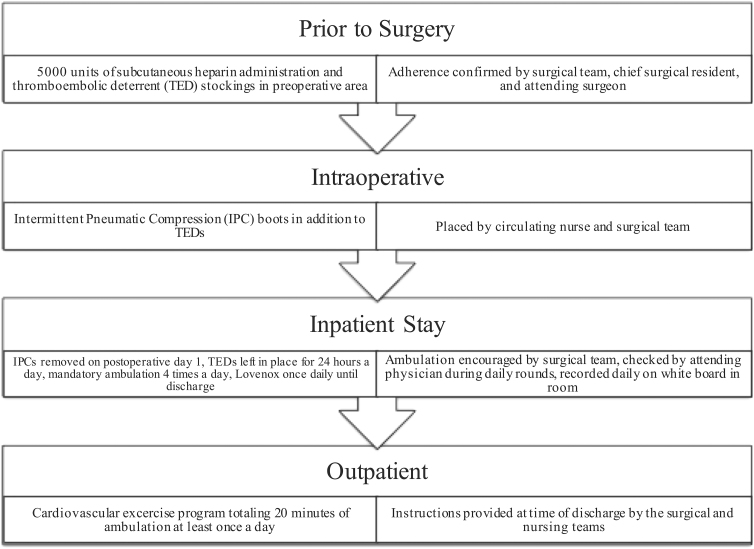
HPBS VTE prevention pathway. Overview of the interventions (on the left) and oversight measures (on the right) employed preoperatively, intraoperatively, and postoperatively in the inpatient and outpatient settings. HPBS, hepatopancreaticobiliary surgery service; VTE, venous thromboembolism.

Our surgical department is a member of the American College of Surgeons National Surgical Quality Improvement Program (ACS NSQIP), which releases semiannual rankings of each participating hospital and service on the basis of adverse event rates. Our institution's hepatopancreaticobiliary surgery service (HPBS), which primarily performs pancreatic surgical procedures, has followed a robust and aggressive VTE prophylaxis pathway (Fig. 1) that has remained largely unchanged over the past 10 years. The HPBS has historically been a low outlier in symptomatic VTE events as determined by the ACS NSQIP, consistently performing in the more favorable bottom decile of all participating institutions.

Given the perceived low rate of symptomatic VTE in the HPBS as suggested by NSQIP semiannual reports, we sought to determine whether the low VTE rate in the HPBS patients is due to the specific interventions implemented by the HPBS, or underlying differences in preoperative patient characteristics. To achieve this goal, we used a cohort-matched multivariate analysis, comparing the NSQIP participant user file (PUF) and our institutional data for a 6-year period.

## Materials and Methods

### Identification of patient populations

After acquiring institutional review board approval, we queried the ACS NSQIP PUF from 2011 to 2016 and our institutional data for the HPBS during the same time period for all patients undergoing pancreatic procedures as captured by current procedural terminology (CPT) codes 48140, 48145, 48150, 48153, 48154, and 48155.^*^ As our institutional data are reported to the ACS for inclusion in the NSQIP PUF, we were able to identify institutional observations within the PUF and mark them as such for the purpose of comparing HPBS and non-HPBS cases. HPBS cases were identified in the PUF by using our institutional data to find observations with perfect matches in multiple variables.

### Creation of the matched cohort

Owing to the likelihood of selection bias in a retrospective study such as this, we used a matching algorithm to create HPBS and national cohorts with equivalent patient populations as determined by multiple preoperative factors (as given in Table 1). To achieve this, we applied a coarsened exact matching procedure, which coarsens the data through the creation of meaningful strata, and then attempts to match each observation.^21^ The matching process was done without replacement. The result is matched cohorts with an equal number of patients with equivalent preoperative parameters in each group (Table 1).

**Table 1.** Preoperative Factors and Comorbidities

Parameter	Aggregate cohort, *n* (%)			Matched cohort, *n* (%)		
	HPBS	NSQIP	*p*	HPBS	NSQIP	*p*
	*N* = 850	*N* = 35,585		*N* = 803	*N* = 803	
Age >75 years	174 (20.5)	5720 (16.1)	0.001^*^	153 (19.1)	153 (19.1)	1.000
Male	436 (51.3)	17,900 (50.3)	0.568	410 (51.1)	410 (51.1)	1.000
Female	414 (48.7)	17,685 (49.7)		393 (48.9)	393 (48.9)	
Black	71 (8.4)	3299 (9.3)	0.361	65 (8.1)	65 (8.1)	1.000
Asian	19 (2.2)	1254 (3.5)	0.043^*^	17 (2.1)	17 (2.1)	1.000
Hawaiian	0 (0.0)	81 (0.2)	0.164	0 (0.0)	0 (0.0)	—
Unknown race	20 (2.4)	3093 (8.7)	<0.001^*^	16 (2.0)	16 (2.0)	1.000
White	679 (79.9)	27,419 (77.1)	0.052	662 (82.4)	662 (82.4)	1.000
Indian	0 (0.0)	119 (0.3)	0.091	0 (0.0)	0 (0.0)	—
BMI <18	16 (1.9)	1081 (3.0)	0.051	14 (1.7)	14 (1.7)	1.000
BMI 25–30	528 (62.1)	22,583 (63.5)	0.421	501 (62.4)	501 (62.4)	1.000
BMI >30	239 (28.1)	10,424 (29.3)	0.457	227 (28.3)	227 (28.3)	1.000
Transferred from non-home facility	20 (2.4)	1122 (3.2)	0.186	9 (1.1)	9 (1.1)	1.000
Diabetes	221 (26.0)	9040 (25.4)	0.693	205 (25.5)	205 (25.5)	1.000
Current smoker	130 (15.3)	7017 (19.7)	0.001^*^	117 (14.6)	117 (14.6)	1.000
Dyspnea	80 (9.4)	2138 (6.0)	<0.001^*^	65 (8.1)	65 (8.1)	1.000
Functional status	1 (0.1)	317 (0.9)	0.008^*^	1 (0.1)	1 (0.1)	1.000
On a ventilator	1 (0.1)	45 (0.1)	0.943	0 (0)	0 (0)	—
COPD	15 (1.8)	1623 (4.6)	<0.001^*^	13 (1.6)	13 (1.6)	1.000
Ascites	0 (0.0)	144 (0.4)	0.063	0 (0.0)	0 (0.0)	—
CHF	0 (0.0)	137 (0.4)	0.070	0 (0.0)	0 (0.0)	—
HTN	474 (55.8)	18,877 (53.0)	0.117	449 (55.9)	449 (55.9)	1.000
Renal failure	0 (0.0)	47 (0.1)	0.289	0 (0.0)	0 (0.0)	—
On dialysis	3 (0.4)	149 (0.4)	0.769	0 (0.0)	0 (0.0)	—
Preoperative wound infection	2 (0.2)	223 (0.6)	0.150	1 (0.1)	1 (0.1)	1.000
Steroid use	16 (1.9)	1065 (3.0)	0.059	11 (1.4)	11 (1.4)	1.000
Recent weight loss	120 (14.1)	4702 (13.2)	0.442	106 (13.2)	106 (13.2)	1.000
Bleeding disorder	15 (1.8)	1082 (3.0)	0.031^*^	11 (1.4)	11 (1.4)	1.000
Recent transfusion	4 (0.5)	437 (1.2)	0.046^*^	0 (0.0)	0 (0.0)	—
Preoperative sepsis	1 (0.1)	575 (1.6)	0.001^*^	0 (0.0)	0 (0.0)	—
Emergency procedure	1 (0.1)	246 (0.7)	0.044^*^	0 (0.0)	0 (0.0)	—
ASA >2	592 (69.6)	26,025 (73.1)	0.024^*^	561 (69.9)	561 (69.9)	1.000
Disseminated cancer	19 (2.2)	1917 (5.4)	<0.001^*^	17 (2.1)	17 (2.1)	1.000

^*^ Indicates a significant result.

ASA, American Society of Anesthesiologists; BMI, body mass index; CHF, congestive heart failure; COPD, chronic obstructive pulmonary disease; HPBS, hepatopancreaticobiliary surgery service; HTN, hypertension; NSQIP, National Surgical Quality Improvement Program.

### Statistical analysis

Statistical analysis was performed using Stata v13 (StataCorp). All analyses were performed on both the aggregate and matched cohorts. VTE was defined as any deep vein thrombosis (DVT) or pulmonary embolus (PE) reported in the PUF. Within NSQIP, a reportable DVT or PE is a new thrombus within 30 days of surgery necessitating treatment. Descriptive and comparative analyses of the HPBS and national cohorts were done using a chi-square test for categorical variables and *t*-test with equal variance for continuous variables. The same method was also used to determine whether or not VTE rates were significantly different between the national cohort and the HPBS patients, as well as to perform a secondary analysis of additional perioperative complications. Univariate analysis of baseline risk factors for VTE was performed using logistic regression to determine odds ratios (ORs), with a *p* < 0.05 being considered statistically significant. Inclusion in the multivariate analysis was reserved for those factors yielding a *p* < 0.1 in the univariate analysis.

## Results

In total, 36,489 pancreatic operations were identified in the 2011–2016 ACS NSQIP PUFs. Within our institution, the HPBS performed operations on 1046 patients in that same time period, of which 853 (82.0%) were coded as a pancreatic excision. Of those patients, 850 (99.6%) were successfully identified within the PUF, accounting for 2.3% of the pancreatic operations reported nationally. Of note, no VTE event occurred in any of the three unidentified observations. During the identification of the HPBS patients within the PUF, 54 (0.1%) national observations were excluded to optimize the identification process. This resulted in 36,435 total cases (850 HPBS and 35,585 national).

Overall, 1266 patients (3.4%) had a reported symptomatic VTE event; the HPBS had a significantly lower rate of symptomatic VTE than the national cohort (*p* = 0.018); 17/850 (2.0%) and 1249/35,585 (3.5%), respectively (Table 2). As given in Table 3, in the aggregate cohort, univariate analysis identified 17 parameters significantly associated with VTE (*p* < 0.05). To account for the confounding effects that each variable can have on outcomes, a multivariate logistic regression was run, controlling for the 17 variables that achieved statistical significance in the univariate analysis as well as two additional variables that had a p-value of <0.1. This analysis (Table 3) indicated that four factors were significantly associated with an elevated likelihood of VTE: age >75 years, body mass index (BMI) >30, history of bleeding disorder, and American Society of Anesthesiologists (ASA) class >2. Conversely, Asian race, current smoking, and procedure performed by the HPBS independently conferred a lower likelihood of developing a VTE postoperatively.

**Table 2.** Intraoperative and Postoperative Parameters

Parameter	Aggregate cohort, *n* (%)			Matched cohort, *n* (%)		
	HPBS	NSQIP	*p*	HPBS	NSQIP	*p*
	*N* = 850	*N* = 35,585		*N* = 803	*N* = 803	
Operative time (minutes; mean ± SD)^a^	397.6 ± 117.5	322.4 ± 142.3	<0.001^*^	397.5 ± 118.0	316.9 ± 137.0	<0.001^*^
Total hospital LOS (days; mean ± SD)^a^	8.3 ± 6.2	10.8 ± 9.3	<0.001^*^	8.3 ± 6.3	10.3 ± 7.4	<0.001^*^
Wound infection	155 (18.2)	7217 (20.3)	0.142	148 (18.4)	161 (20.0)	0.411
Superficial	39 (4.6)	2533 (7.1)	0.004^*^	35 (4.4)	52 (6.5)	0.079
Deep	6 (0.7)	737 (2.1)	0.005^*^	6 (0.7)	18 (2.2)	0.014^*^
Organ/space	117 (13.8)	4335 (12.2)	0.164	113 (14.1)	99 (12.3)	0.302
Dehiscence	1 (0.1)	433 (1.2)	0.004^*^	1 (0.1)	8 (1.0)	0.019^*^
Respiratory complication	27 (3.2)	2305 (6.5)	<0.001^*^	25 (3.1)	46 (5.7)	0.011^*^
Pneumonia	18 (2.1)	1466 (4.1)	0.004^*^	17 (2.1)	23 (2.9)	0.337
Ventilator dependence	16 (1.9)	1164 (3.3)	0.024^*^	15 (1.9)	20 (2.5)	0.393
Reintubation	17 (2.0)	1334 (3.7)	0.008^*^	16 (2.0)	32 (4.0)	0.019^*^
VTE^b^	17 (2.0)	1249 (3.5)	0.018^*^	17 (2.1)	31 (3.9)	0.040^*^
DVT	11 (1.3)	923 (2.6)	0.052	11 (1.4)	23 (2.9)	0.096
PE	7 (0.8)	437 (1.2)	0.288	7 (0.9)	13 (1.6)	0.177
Time to VTE formation (days; mean ± SD)^a^	17.1 ± 5.0	13.2 ± 8.0	0.105	17.1 ± 5.0	11.9 ± 5.9	0.019^*^
Cardiac arrest	2 (0.2)	391 (1.1)	0.016^*^	2 (0.2)	6 (0.7)	0.156
Myocardial infarction	1 (0.1)	367 (1.0)	0.008^*^	1 (0.1)	8 (1.0)	0.019^*^
Sepsis	61 (7.2)	2854 (8.0)	0.370	58 (7.2)	54 (6.7)	0.695
Reoperation	19 (2.2)	1875 (5.3)	<0.001^*^	17 (2.1)	43 (5.4)	0.001^*^
Readmission	10 (1.2)	639 (1.8)	0.055	10 (1.2)	18 (2.2)	0.079

^a^ Calculated using *t*-test of equal variance.

^b^ In the aggregate, 1 patient in the HPBS cohort and 111 patients in the NSQIP cohort had both a DVT and PE; in the matched data set, 1 patient in the HPBS cohort and 5 patients in the NSQIP cohort had both a DVT and PE.

^*^ Indicates a significant result.

DVT, deep vein thrombosis; LOS, length of stay; PE, pulmonary embolus; SD, standard deviation; VTE, venous thromboembolism.

**Table 3.** Univariate and Multivariate Analysis of Aggregate Cohort Venous Thromboembolism Risk

Parameter	Univariate (*n* = 36,435)			Multivariate (*n* = 36,435)		
	OR	95% CI	*p*	OR	95% CI	*p*
Age >75 years	1.247	1.082–1.438	0.002^*^	1.223	1.055–1.418	0.007^*^
Sex	1.151	1.029–1.288	0.014^*^	1.117	0.997–1.252	0.057
Black	0.970	0.798–1.180	0.760	—	—	—
Asian	0.390	0.244–0.623	<0.001^*^	0.485	0.299–0.789	0.004^*^
Hawaiian	1.069	0.337–3.390	0.910	—	—	—
Unknown race	0.905	0.735–1.115	0.348	—	—	—
White	1.239	1.076–1.427	0.003^*^	1.126	0.972–1.305	0.113
Indian	0.718	0.228–2.261	0.571	—	—	—
BMI <18 kg/m^2^	1.081	0.787–1.485	0.629	—	—	—
BMI 25–30 kg/m^2^	1.379	1.220–1.559	<0.001^*^	1.122	0.972–1.296	0.117
BMI >30 kg/m^2^	1.509	1.344–1.694	<0.001^*^	1.294	1.216–1.598	<0.001^*^
Transferred from non-home facility	1.228	0.914–1.648	0.173	—	—	—
Diabetes	1.098	0.968–1.246	0.145	—	—	—
Current smoker	0.781	0.671–0.909	0.001^*^	0.815	0.698–0.953	0.010^*^
Dyspnea	1.086	0.865–1.362	0.478	—	—	—
Functional status >1	1.588	1.008–2.500	0.046^*^	1.280	0.806–2.033	0.295
On a ventilator	3.397	1.340–8.612	0.010^*^	1.675	0.593–4.728	0.330
COPD	1.215	0.947–1.559	0.127	—	—	—
Ascites	2.082	1.092–3.968	0.026^*^	1.668	0.861–3.231	0.130
CHF	2.197	1.151–4.192	0.017^*^	1.666	0.865–3.207	0.127
HTN	1.095	0.979–1.226	0.114	—	—	—
On dialysis	1.142	0.504–2.589	0.750	—	—	—
Wound infection	1.571	0.876–2.817	0.130	—	—	—
Steroid use	1.302	0.970–1.749	0.079	1.215	0.903–1.635	0.198
Recent weight loss	0.871	0.732–1.036	0.118	—	—	—
Bleeding disorder	1.698	1.306–2.208	<0.001^*^	1.482	1.136–1.934	0.004^*^
Recent transfusion	1.829	1.234–2.711	0.003^*^	1.412	0.920–2.169	0.115
Preoperative sepsis	1.429	0.973–2.098	0.068	1.066	0.696–1.632	0.770
Emergency procedure	1.936	1.163–3.223	0.011^*^	1.442	0.816–2.548	0.208
ASA >2	1.503	1.307–1.729	<0.001^*^	1.366	1.184–1.575	<0.001^*^
Disseminated cancer	1.279	1.020–1.603	0.033^*^	1.229	0.979–1.543	0.076
Procedure performed by HPBS	0.561	0.346–0.910	0.019^*^	0.572	0.352–0.928	0.024^*^

Renal failure was not included in the univariate or multivariate analysis as there were no events noted in the HPBS cohort.

— indicates that a variable was not included in multivariate analysis.

^*^ Indicates a significant result.

CI, confidence interval; OR, odds ratio.

**FIG. 2.**
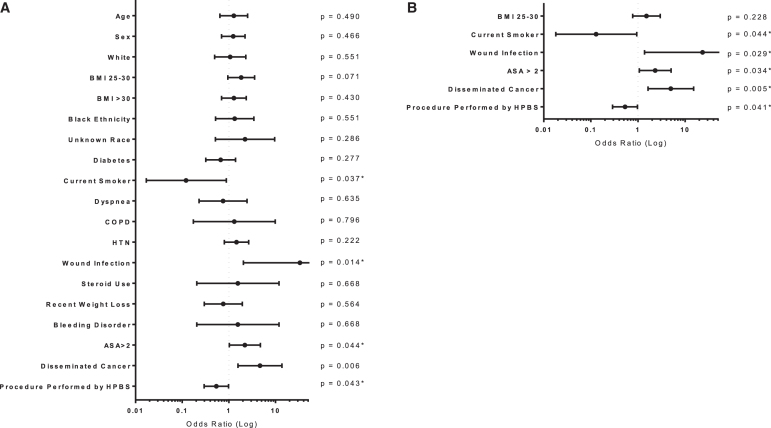
Analysis of VTE risk in the matched cohort. **(A)** Univariate, **(B)** multivariate. *Indicates a significant result. ASA, American Society of Anesthesiologists; BMI, body mass index; CHF, congestive heart failure; COPD, chronic obstructive pulmonary disease; HPBS, hepatopancreaticobiliary surgery service; HTN, hypertension.

The matching algorithm yielded 803 patients in each cohort for analysis. Once again, the rate of symptomatic VTE was lower in the HPBS group than nationally, at 17/803 (2.1%) and 31/803 (3.9%), respectively (*p* = 0.040). We next assessed the influence of preoperative factors and operating facility on likelihood of VTE using a univariate logistic regression (Fig. 2A), which identified six factors to be included in the multivariate regression. As shown in Figure 2B, all but one of these parameters was shown to be an independent predictor of VTE incidence; preoperative wound infection (OR = 23.386, *p* = 0.029), ASA class >2 (OR = 2.316, *p* = 0.034), and disseminated cancer (OR = 4.960, *p* = 0.005) conferred a higher likelihood of symptomatic VTE, whereas current smoking (OR = 0.129, *p* = 0.044) and procedure performed by the HPBS (OR = 0.530, *p* = 0.041) had a protective influence.

### Secondary analysis

Although not the original focus of this study, a comparison of non-VTE outcomes yielded interesting results. As given in Table 2, the mean total hospital LOS was significantly lower for patients operated on by the HPBS than the national population in both the matched and aggregate cohorts (aggregate: 8.3 ± 6.2 vs. 10.8 ± 9.3 days, *p* < 0.001; matched: 8.3 ± 6.3 vs. 10.3 ± 7.4, *p* < 0.001). Furthermore, patients in the HPBS cohort had a significantly lower reoperation rate (aggregate: *p* < 0.001; matched: *p* = 0.001). In addition, the rate of overall respiratory complications was significantly lower in the HPBS cohort (*p* < 0.001 in the aggregate, *p* = 0.011 in the matched), as was the rate of myocardial infarction (*p* = 0.008 in the aggregate, *p* = 0.019 in the matched). Deep surgical site infections (SSIs) and wound dehiscence were also significantly lower in both the aggregate and matched HPBS groups. Cardiac arrest and superficial SSI were lower in the HPBS group in the aggregate, but this significance was not confirmed upon matching.

## Discussion

It has long been suggested that appropriate prescribing patterns and proper adherence to thromboembolism prophylaxis reduce the rate of VTE. Multimodal prophylaxis has been studied extensively in orthopedics and other surgical fields, although to our knowledge no study in pancreatic surgery has previously shown an independent effect of a prophylactic pathway on rates of symptomatic VTE.^22,23^ Understanding the potential reasons underlying national low outliers in VTE rates has great potential for informing patient care and improving hospital practice.

Our results demonstrate a statistically significant lower rate of VTE events in the HPBS patients than national rates. We sought to determine whether or not the prophylactic pathway of the HPBS may play a role in the low rates of symptomatic VTE seen in those patients. To do so, it was necessary to determine the independent risk factors associated with VTE occurrence in both the HPBS and nationally, and to further determine whether those factors are confounded in any way. We were able to identify 850 HPBS patients in the NSQIP PUF, which afforded us the opportunity to effectively compare HPBS patients with the national subset. Although many factors appeared to be contributors to VTE risk, upon multivariate analysis, it was found that only older age, high BMI, history of a bleeding disorder, and high ASA class independently increase the risk of VTE. Furthermore, we found that Asian race, being a current smoker, and having the procedure performed by the HPBS conferred a significantly lower rate of VTE events. It is unclear why current smoking status has a protective effect, as smoking is a known risk factor for VTE formation.^18^ That said, VTE risk indices, including the Caprini Risk Index and the Geerts Index, do not include smoking as a stratifying risk factor, suggesting that other factors, such as length of surgery, active cancer, and BMI, are more influential on VTE formation.^17,18^

Taken together, the results suggest that the difference in VTE rates between the HPBS and national cohorts is not due to patient factors alone and is likely a result of processes of care provided at our institution. In contrast to many other surgical services, all HPBS patients receive the same prophylaxis, which is designed for high-risk individuals, regardless of predisposing risk factors. This is an appropriate method given the high prevalence of VTE risk factors in this patient population (such as extended length of surgery, older age, and malignancy).

The strict VTE prevention pathway (Fig. 1), which the HPBS has followed for the length of the study period, consists of carefully timed utilization of anticoagulation, TED stockings, IPC boots, and ambulation. Uniquely, the HPBS uses both IPCs and TEDs for mechanical prophylaxis in the first 24 h after surgery but discontinues the use of IPCs in favor of TEDs alone for the rest of the hospital stay. The decision to use TED stockings during the entire hospital stay was made by the attending surgeons on the service due to an anecdotally higher adherence than with IPCs; this is a novel practice at our institution. Potentially the most distinctive portion of the HPBS protocol is the use of rotating third-year medical students to encourage patients to meet ambulation goals during their hospital stay (four times a day for 200 feet). The students aid the patients in ambulating and record the ambulation event in the patient's room. A hard copy of the number of ambulation events is reviewed by the attending surgeon for each patient at the end of the day. As a result of these measures, the HPBS vastly outperforms other surgical and medical services at our hospital in terms of ambulation adherence, despite having frailer patients. We present the VTE prevention model put forth by the HPBS so that it may serve as a framework for surgical services at other institutions that aim to decrease VTE rates.

A current matter of debate relates to extension of chemoprophylaxis after discharge. During the study period, we did not routinely utilize extended chemoprophylaxis. Guidelines from CHEST released in 2012 suggested extending chemoprophylaxis for 30 days after surgery in cancer patients who are high risk for forming VTE and for whom there is no contraindication to anticoagulation, but there is no recommendation for patients undergoing abdominal surgery for reasons other than malignancy.^24^ There is still a paucity of evidence regarding extended chemoprophylaxis for VTE prevention in noncancer patients.

Our secondary analysis found that the HPBS patients had a significantly shorter hospital LOS than non-HPBS patients. This may be due to the lower reoperation rates, the lower rates of postoperative complications (including symptomatic VTE), or simply hospital- and service-specific discharge practices. Given the increased negative impact that long periods of immobilization have on VTE formation, the shorter LOS seen in the HPBS patients could be a contributor to the decreased rates of VTE. In addition, a larger proportion of VTEs occurred before discharge in the national cohort than in the HPBS cohort, which may be because non-HPBS patients spend, on average, more time in the hospital, and thus have a greater potential for developing VTE (Table 2).

Although the main focus of this project was to compare the symptomatic VTE rate between the HPBS and the national cohort, many other postoperative complications were found to occur at a lower rate in the HPBS patients, including superficial and deep SSI, wound dehiscence, respiratory complications, and myocardial infarctions. Although not described here, the HPBS has additional protocols for prevention of these adverse events. The lower rates of these specific complications may also be influencing LOS and, therefore, lowering the risk of VTE for HPBS patients. In addition, it has been shown that early ambulation can reduce the risk of postoperative respiratory and cardiac complications, so the HPBS pathway may, in addition to event-specific prophylaxis, account for these lower rates.

### Limitations

There are several limitations of this study that need to be acknowledged. The retrospective nature of this study prevents us from speaking to causation, and as such we can only make correlative assessments and conclusions. As with any large database review such as this, there are many potential confounders that are not measured that could influence the outcomes of the study, including cancer metrics, nonsurgical treatments, and use of anticoagulation or antiplatelet therapy. However, the deidentified nature of the NSQIP database precludes more granular data acquisition. Similarly, the NSQIP PUF is an imperfect database due to selective inclusion and reporting criteria. Not all pancreatic cases in the nation are reported in NSQIP, which introduces both selection bias and confounding. Furthermore, adverse events, including symptomatic DVT and PE, are only captured within 30 days of operation, and there is no formal use of routine lower extremity ultrasonography for the detection of DVT or reporting of incidentally discovered VTEs, potentially leading to missed incidences. It is important to note that NSQIP does not capture the prophylactic modalities that were offered to each patient; therefore, it is impossible to determine whether the patients at other institutions received similar or dissimilar inpatient prophylactic regimens as compared with the HPBS patients. Reporting and sharing these prophylactic process measures would be an incredibly powerful tool for guiding institutions in identifying which areas need improvement to reduce rates of VTE.

One line of inquiry that we did not pursue was the consideration of VTE in patients undergoing open versus minimally invasive procedures. Information regarding surgical approach is not included in the main NSQIP PUF, but it is included in the recently implemented pancreas site-specific PUF. This site-specific file is not available for the entirety of the study period, and confining our study to just those years would have excluded two-thirds of our patient population. As such, although we think that this would have been a helpful variable to include, it was not feasible for our cohort.

To identify patients undergoing a pancreatic surgical procedure, we used CPT billing codes. There are inherent limitations to using these codes, as billing procedures are not consistent across institutions, and miscoding may occur. In addition, when querying the PUF for patients undergoing pancreatic procedures, we used only CPT codes utilized by the HPBS. There are a number of other pancreatic surgery-associated codes that are not used by the HPBS; as such, there may be non-HPBS patients with procedures similar to the HPBS cohort whose surgeries were billed under an excluded CPT code, and were, therefore, not included in our study. Although we could have included other CPT codes, we felt that using only HPBS codes would yield the most homogeneous study sample and would ensure that no dissimilar surgeries were included.

## Conclusions

In following the described multimodal prophylaxis pathway, the HPBS has been consistently identified as a low outlier in VTE rates. We have shown that this designation represents a significantly lower rate of VTE events than the national subset of pancreatic procedures captured in the NSQIP PUF over a large time period. Through the utilization of matching and multivariate analyses, we were able to identify an independent protective effect of the HPBS on VTE incidence, which we believe to be due, at least in part, to the VTE prevention pathway employed by the HPBS, which is diligently adhered to and checked by multiple care providers. The shorter hospital LOS of the HPBS patients, as well as the lower rates of respiratory complications, cannot be understated as well. Further prospective studies are warranted to more accurately determine the parameters associated with VTE events in both the HPBS patients and pancreatic surgery patients at other institutions.

## Abbreviations Used

ACS: American College of Surgeons
ASA: American Society of Anesthesiologists
BMI: body mass index
CPT: current procedural terminology
DVT: deep vein thrombosis
HPBS: hepatopancreaticobiliary surgery service
IPC: intermittent pneumatic compression
LOS: length of stay
NSQIP: National Surgical Quality Improvement Program
OR: odds ratio
PE: pulmonary embolus
PUF: participant user file
SSIs: surgical site infections
TED: thromboembolic deterrent
VTE: venous thromboembolism

## Notes

^*^ These codes represent excision procedures of the pancreas.

## References

[1] Office of the Surgeon General, National Heart, Lung, and Blood Institute. The surgeon general's call to action to prevent deep vein thrombosis and pulmonary embolism. Rockville, MD: Office of the Surgeon General; 2008 20669525

[2] Schleyer AM, Robinson E, Dumitru R, et al. Preventing hospital-acquired venous thromboembolism: improving patient safety with interdisciplinary teamwork, quality improvement analytics, and data transparency. J Hosp Med. 2016;11 Suppl 2:S38–S43 27925422 10.1002/jhm.2664

[3] Alikhan R, Peters F, Wilmott R, et al. Fatal pulmonary embolism in hospitalised patients: a necropsy review. J Clin Pathol. 2004;57:1254–1257 15563663 10.1136/jcp.2003.013581 PMC1770519

[4] Tufano A, Coppola A, Cerbone AM, et al. Preventing postsurgical venous thromboembolism: pharmacological approaches. Semin Thromb Hemost. 2011;37:252–266 21455859 10.1055/s-0031-1273089

[5] Collins R, Scrimgeour A, Yusuf S, et al. Reduction in fatal pulmonary embolism and venous thrombosis by perioperative administration of subcutaneous heparin. Overview of results of randomized trials in general, orthopedic, and urologic surgery. N Engl J Med. 1988;318:1162–1173 3283548 10.1056/NEJM198805053181805

[6] Koch A, Bouges S, Ziegler S, et al. Low molecular weight heparin and unfractionated heparin in thrombosis prophylaxis after major surgical intervention: update of previous meta-analyses. Br J Surg. 1997;84:750–759 9189079

[7] Almutairi AR, Zhou L, Gellad WF, et al. Effectiveness and safety of non-vitamin k antagonist oral anticoagulants for atrial fibrillation and venous thromboembolism: a systematic review and meta-analyses. Clin Ther. 2017;39:1456–1478.e36. 28668628 10.1016/j.clinthera.2017.05.358

[8] Agu O, Hamilton G, Baker D Graduated compression stockings in the prevention of venous thromboembolism. Br J Surg. 1999;86:992–1004 10460633 10.1046/j.1365-2168.1999.01195.x

[9] Ho KM, Tan JA Stratified meta-analysis of intermittent pneumatic compression of the lower limbs to prevent venous thromboembolism in hospitalized patients. Circulation. 2013;128:1003–1020 23852609 10.1161/CIRCULATIONAHA.113.002690

[10] Adogwa O, Elsamadicy AA, Fialkoff J, et al. Early ambulation decreases length of hospital stay, perioperative complications and improves functional outcomes in elderly patients undergoing surgery for correction of adult degenerative scoliosis. Spine. 2017;42:1420–1425 28902101 10.1097/BRS.0000000000002189

[11] Khandhar S, Powers C, Schatz C, et al. Early post-operative ambulation after thoracic surgery—the WAVE experience. J Thorac Oncol. 2017;12:S244–S245

[12] Pearse EO, Caldwell BF, Lockwood RJ, et al. Early mobilisation after conventional knee replacement may reduce the risk of postoperative venous thromboembolism. J Bone Joint Surg Br. 2007;89:316–322 17356141 10.1302/0301-620X.89B3.18196

[13] Olszewski T, Noonan K, Gold H, et al. Ambulation protocols leading to decreased postoperative complications and hospital stay. House Staff Qual Improv Patient Saf Posters. 2017 Available at http://jdc.jefferson.edu/patientsafetyposters/43 Accessed 18, 2020

[14] Miwa S, Visintainer P, Engelman R, et al. Effects of an ambulation orderly program among cardiac surgery patients. Am J Med. 2017;130:1306–1312 28551042 10.1016/j.amjmed.2017.04.044 PMC6004606

[15] Teodoro CR, Breault K, Garvey C, et al. STEP-UP: study of the effectiveness of a patient ambulation protocol. Medsurg Nurs. 2016;25:111–116 27323470

[16] Cassidy MR, Rosenkranz P, McAneny D Reducing postoperative venous thromboembolism complications with a standardized risk-stratified prophylaxis protocol and mobilization program. J Am Coll Surg. 2014;218:1095–1104 24768293 10.1016/j.jamcollsurg.2013.12.061

[17] Geerts WH, Bergqvist D, Pineo GF, et al. Prevention of venous thromboembolism: American College of Chest Physicians evidence-based clinical practice guidelines (8th edition). Chest. 2008;133(6 Suppl):381S-453S 18574271 10.1378/chest.08-0656

[18] Caprini JA Thrombosis risk assessment as a guide to quality patient care. Dis Mon. 2005;51:70–78 15900257 10.1016/j.disamonth.2005.02.003

[19] Barbar S, Noventa F, Rossetto V, et al. A risk assessment model for the identification of hospitalized medical patients at risk for venous thromboembolism: the Padua Prediction Score. J Thromb Haemost. 2010;8:2450–2457 20738765 10.1111/j.1538-7836.2010.04044.x

[20] Liu X, Liu C, Chen X, et al. Comparison between Caprini and Padua risk assessment models for hospitalized medical patients at risk for venous thromboembolism: a retrospective study. Interact Cardiovasc Thorac Surg. 2016;23:538–543 27297558 10.1093/icvts/ivw158

[21] Blackwell M, Iacus S, King G, et al. Cem: coarsened exact matching in Stata. Stata J. 2009;9:524–546

[22] González Della Valle A, Serota A, Go G, et al. Venous thromboembolism is rare with a multimodal prophylaxis protocol after total hip arthroplasty. Clin Orthop. 2006;444:146–153 16446593 10.1097/01.blo.0000201157.29325.f0

[23] Knesek D, Peterson TC, Markel DC Thromboembolic prophylaxis in total joint arthroplasty. Thrombosis. 2012;2012:837896 23029611 10.1155/2012/837896 PMC3458274

[24] Gould MK, Garcia DA, Wren SM, et al. Prevention of VTE in nonorthopedic surgical patients. Chest. 2012;141(2 Suppl):e227S–e277S 22315263 10.1378/chest.11-2297 PMC3278061

